# Evaluation of a training program for Italian general practitioners to improve substance abuse early identification and brief intervention and identification of cardiovascular risk

**DOI:** 10.1186/1940-0640-7-S1-A85

**Published:** 2012-10-09

**Authors:** Pierluigi Struzzo, Luigi Canciani, Alberto G Barsanti

**Affiliations:** 1 Regional Center for Training in Primary Care, Monfalcone, Italy

## 

General practitioners (GPs) often cite inadequate training as one of the reasons for not performing early identification and brief intervention (EIBI) for substance use disorders. Providing “more” medical education on EIBI is an important step for policy makers. From an economic and public health point of view, it is important to know how much is “more.” We summarize the results of a one-day continuing medical education (CME) training of Italian GPs on EIBI, unhealthy lifestyles, and cardiovascular risk. An eight-hour CME interdisciplinary training day was offered to the 1040 GPs in the Udine region of Italy. Lessons by expert GPs, cardiologists, and public health professionals were offered on CV risk reduction by provision of EIBI and brief motivational interviewing to address unhealthy lifestyles. Video role-playing presentations were shown and discussed in the afternoon session. Of the 774 GPs who attended the training day, 563 (72.7%) completed a pre- and post-training questionnaire assessing EIBI general knowledge, self-efficacy, and professional satisfaction level using 11-point (0-10) scales. We analyzed the correlation between pre- and post-training self-efficacy on different lifestyles counseling using the Wilcoxon Mann-Whitney test and the correlation between professional satisfaction level and self-efficacy on lifestyles counseling using the Spearman Rank Correlation Coefficient. After training, a significant improvement in general knowledge and self efficacy were demonstrated by most of the participants (p = 0.0001) . Pre- and post-training differences were all significant, but GPs showed less self-efficacy on addressing alcohol use (p = 0.5902) compared with addressing tobacco use (p = 0.8961), weight control (p = 0.3008), and physical activity (p = 0.4054). Self-efficacy was linked to professional satisfaction level. One day of interdisciplinary CME training on EIBI and cardiovascular risk reduction increased GPs’ self-efficacy, but professional satisfaction needs to also be addressed in future training programs. Practical implementation should follow a short time after training so as not to reduce the effect.

